# Editorial: Prosocial and hypersocial behavior: From genes to circuits and behavior

**DOI:** 10.3389/fnbeh.2022.1090151

**Published:** 2022-12-02

**Authors:** Miklos Toth

**Affiliations:** Department of Pharmacology, Weill Cornell Medical College, New York, NY, United States

**Keywords:** social behavior, prosociability, hypersociability, genes, circuits

Impaired and abnormal social behavior has been extensively studied as it is often associated with autism spectrum disorder, anxiety, and anti-social personality disorder. Opposite manifestations of social behavior, such as an excessive need for social contact and emotional dependence on continuous social company and attention, have also been recognized as potentially abnormal but are not as well-understood.

As with many behaviors, increased sociability exists on a continuum ranging from prosociability to the more extreme hypersociability (Toth, 2019). Prosocial personality is considered advantageous in a society; however, hypersociability is often inappropriate and is frequently associated with neurodevelopmental disorders or maltreatment during childhood. In this Research Topic of Frontiers in Behavioral Neuroscience, select themes on prosociability and hypersociability in both humans and animals are explored from genes to circuits and behavior.

Twin studies suggest that prosocial behavior has a genetic basis (Rushton, 2004). Similarly, pathological hypersociability can be genetic, including conditions that are caused by chromosomal deletions and monogenic mutations (Morris and Mervis, 2000). One such condition is Angelman Syndrome (AS), a rare genetic disorder that involves disruption to the *UBE3A* gene on the maternal chromosome by mutation, *de novo* deletion, imprinting defect or paternal uniparental disomy. Characteristic features of AS include motivation for social contact and seeking of sensory stimuli. A paper by Heald et al. in the present series reports the development of an experimental paradigm to quantify these features and finds that children with a mutation were more likely to be reinforced by social stimuli than children with a deletion in *UBE3A*. These data indicate that not all children with AS exhibit a strong drive for social contact and that success of reward-based intervention may be dependent on the genetic subtype of AS.

Prosocial and hypersocial behavior is not limited to human sociability; indeed, it is observed in various social species, including non-human primates (de Waal and Suchak, 2010), dogs (vonHoldt et al., 2017), but also rodents (Langford et al., 2006), suggesting the existence of evolutionary conserved mechanisms. However, there is variability in social interactions between species, from preference of novel conspecifics to favoring familiar conspecifics, that may help to better understand the genetic and neurobiological mechanisms underlying the various forms of social behavior. Beery and Shambaugh assessed such variability in familiarity preferences in five rodent species that each live in groups but have different social systems. They found high within-group consistency but species-specific divergence in social behavior; preference for familiarity in prairie voles and meadow voles but a preference for social novelty in rats, mice and degus. Since behaviors tend to vary with ambient conditions, high consistency in preference within groups through years indicates different phylogenetic traits underlying the relatively closed social structure of voles and flexible group structure and gregariousness of the other species.

Although pro/hypersocial behavior is determined by genetic factors and environmentally induced epigenetic programs, it is ultimately neuronal circuit (dys)function that leads to its behavioral manifestation. The circuit governing social behavior includes the prefrontal cortex, amygdala, and hippocampus, among other brain regions. Synaptic communication between these regions is crucial in social behavior and components of the synaptic machinery have been linked to hypersociability. Postsynaptic density protein-95 (PSD-95) is an abundant synaptic scaffolding protein that facilitates synaptic maturation by recruiting glutamate receptors. Gao and Mack report that a deficit in PSD95 results in hypersociability in adulthood that was preceded by hyposocial behavior during adolescence. Because of the prolonged maturation of the medial prefrontal cortex, an essential region in social behavior during adolescence, the authors speculate that PSD95 deficit disrupts circuit development that, perhaps indirectly, leads to hypersociability later in life.

Importance of the adolescent period in social behavior is also emphasized by Yoon et al. who examined how brain functional connectivity may contribute to personality dimensions related to social behavior in adolescence. Using fMRI, the authors found that functional connectivity of the amygdala with various regions of the social network is associated with sex-specific and individual differences in extraversion and agreeableness—two personality dimensions linked to peer acceptance, social network size and friendship quality. These findings were also confirmed with whole brain connectivity analysis.

Brain functional connectivity is further explored by Liu et al. Previous findings suggest that songs with neutral lyrics increase prosocial behavior in humans such as prosocial thoughts, interpersonal empathy, and fostered helping behavior (Greitemeyer, 2009). In their investigation of the influence of absolute (*i.e*., non-representational) music on moral judgment to accept or refuse unfair offers, the authors found that music increased the rejection of unfair offers. It also elicited stronger activation of rewarding circuits, including the ventral striatum, in resting EEG. These data are in line with research indicating that music has the ethical power to influence moral judgments and thus, social behavior.

Altogether, the findings put forward in this Research Topic contribute to a better understanding of the neurobiological mechanisms involved in the development of pro/hypersocial behavior.

## Author contributions

The author confirms being the sole contributor of this work and has approved it for publication.

## Funding

This work was supported by US National Institute of Health grant 1RO1MH080194 to MT.

## Conflict of interest

The author declares that the research was conducted in the absence of any commercial or financial relationships that could be construed as a potential conflict of interest.

## Publisher's note

All claims expressed in this article are solely those of the authors and do not necessarily represent those of their affiliated organizations, or those of the publisher, the editors and the reviewers. Any product that may be evaluated in this article, or claim that may be made by its manufacturer, is not guaranteed or endorsed by the publisher.
